# ROS Balance Autoregulating Core–Shell CeO_2_@ZIF-8/Au Nanoplatform for Wound Repair

**DOI:** 10.1007/s40820-024-01353-0

**Published:** 2024-03-21

**Authors:** Xi Zhou, Quan Zhou, Zhaozhi He, Yi Xiao, Yan Liu, Zhuohang Huang, Yaoji Sun, Jiawei Wang, Zhengdong Zhao, Xiaozhou Liu, Bin Zhou, Lei Ren, Yu Sun, Zhiwei Chen, Xingcai Zhang

**Affiliations:** 1 The Higher Educational Key Laboratory for Biomedical Engineering of Fujian Province, Research Center of Biomedical Engineering of Xiamen, Department of Biomaterials, College of Materials, Xiamen University, Xiamen, 361005 People’s Republic of China; 2 Department of Electronic Science, Fujian Provincial Key Laboratory of Plasma and Magnetic Resonance Research, School of Electronic Science and Engineering, Xiamen University, Xiamen, 361005 People’s Republic of China; 3 John A Paulson School of Engineering and Applied Sciences, Harvard University, Cambridge, MA 02138 USA; 4 Department of Otorhinolaryngology, Union Hospital of Tongji Medical College, Huazhong University of Science and Technology, Wuhan, 430022 People’s Republic of China; 5 NO.1 Middle School Affiliated to Central China Normal University, Wuhan, 430223 People’s Republic of China

**Keywords:** Metal–organic framework (MOF), Reactive oxygen species (ROS), Cerium dioxide, Au nanoparticles, Wound healing

## Abstract

Reactive oxygen species (ROS) plays important roles in living organisms. While ROS is a double-edged sword, which can eliminate drug-resistant bacteria, but excessive levels can cause oxidative damage to cells. A core–shell nanozyme, CeO_2_@ZIF-8/Au, has been crafted, spontaneously activating both ROS generating and scavenging functions, achieving the multi-faceted functions of eliminating bacteria, reducing inflammation, and promoting wound healing. The Au Nanoparticles (NPs) on the shell exhibit high-efficiency peroxidase-like activity, producing ROS to kill bacteria. Meanwhile, the encapsulation of CeO_2_ core within ZIF-8 provides a seal for temporarily limiting the superoxide dismutase and catalase-like activities of CeO_2_ nanoparticles. Subsequently, as the ZIF-8 structure decomposes in the acidic microenvironment, the CeO_2_ core is gradually released, exerting its ROS scavenging activity to eliminate excess ROS produced by the Au NPs. These two functions automatically and continuously regulate the balance of ROS levels, ultimately achieving the function of killing bacteria, reducing inflammation, and promoting wound healing. Such innovative ROS spontaneous regulators hold immense potential for revolutionizing the field of antibacterial agents and therapies.

**Supplementary Information:**

The online version contains supplementary material available at 10.1007/s40820-024-01353-0.

## Highlights

- The innovation of this work mainly lies in the auto-regulation of reactive oxygen species (ROS) balance effective by combination of ROS antibacterial and ROS scavenging anti-inflammatory functions with a core–shell nanoplatform (CeO_2_@ZIF-8/Au), which can not only achieve high antibacterial efficiency, but also promote wound healing, and provide a new idea for the nano-catalytic system in the field of wound healing of bacterial infection.

**Supplementary Information:**

The online version contains supplementary material available at 10.1007/s40820-024-01353-0.

## Graphical abstract

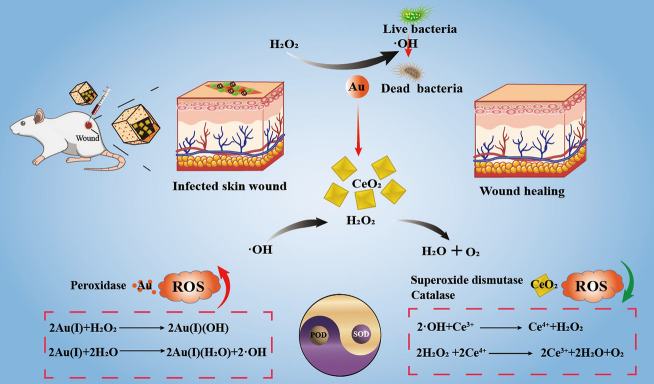

## Introduction

The complex and dynamic nature of reactive oxygen species (ROS) is a source of fascination for scientists and researchers alike. ROS, comprising hydrogen peroxide (H_2_O_2_), superoxide anion (O^2−^), singlet oxygen (^1^O_2_), and hydroxyl radical (·OH), plays crucial roles in living organisms [1–3]. But ROS is a double-edged sword, with its effects varying depending on its concentration and duration of action. At low levels, ROS can function as secondary messengers, aiding in cell signaling pathways, mitotic responses, cell proliferation/migration/differentiation, and resistance to pathogen invasion [4–7]. However, high levels of ROS can disrupt the body's regulatory mechanisms, leading to DNA mutations, cell damage, and the development of related diseases such as aging, cancer, cardiovascular disease, diabetes, ischemic/hypoxic injury, trauma infection, and neurological diseases [8–12]. Therefore, it is critical to modulate ROS levels and keep a balance between ROS generation and scavenging.

Nanozymes, a type of nanomaterial that exhibit high-efficiency catalytic activity similar to enzymes. Among these nanozymes, those that catalyze the in-situ generation of ROS (such as oxidases and peroxidases) have been widely used in anti-tumor and the development of new nano-antibacterial agents [13–19]. Within the tumor microenvironment, ROS can regulate the phenotype and function of tumor cells and various immune cells, affecting multiple processes of tumor immune response [20–24]. This can be applied for tumor-targeting drug delivery systems [25, 26]. Surface-oxidized arsenene nanosheets [27] was fabricated with effective·O^2−^ and ^1^O_2_ generation and glutathione consumption for targeted ROS burst for cancer treatment. In addition, ROS can also destroy multiple vital substances (such as nucleic acids, proteins, and lipids) that are essential for bacterial cell normal physiological activities through oxidative action, and nanozymes can efficiently eliminate drug-resistant bacteria and delay the emergence of bacterial resistance [28–37]. We have synthesized MnFe_2_O_4_@MIL/Au-GOx and ZIF-8/Au-GOx nanozymes that achieve high-efficiency bacterial killing by using a synergistic strategy of cascade catalysis to produce ROS (·OH) and consume glutathione in the bacteria [38, 39]. In contrast, nanozymes with ROS clearance ability, including catalase (CAT), superoxide dismutase (SOD) and glutathione peroxidase (GPx), can mimic the intracellular antioxidant defense system to remove various toxic ROS, and show good therapeutic effects on acute kidney injury (AKI), acute liver injury (AILI), and wound healing and other related diseases [40–46].

Although tremendous progress has been made in generating or removing ROS, balancing ROS levels remains a huge challenge. Recent advances offer exciting possibilities for balancing ROS levels. For example, in photodynamic therapy, pyro pheophytin-bound Mn_3_O_4_ nanozymes can not only remove ROS in normal tissues but also produce ROS in the tumor microenvironment [47]. With the help of PDT or PTT, nanoceria and MoS_2_-CeO_2_ nanomaterials can also be used to enhance antibacterial and antioxidant activity [48, 49]. These advances provide new directions, however, the above process still requires the trigger of an external force (light) to activate the ROS adjustment switch.

**Fig. 1:**
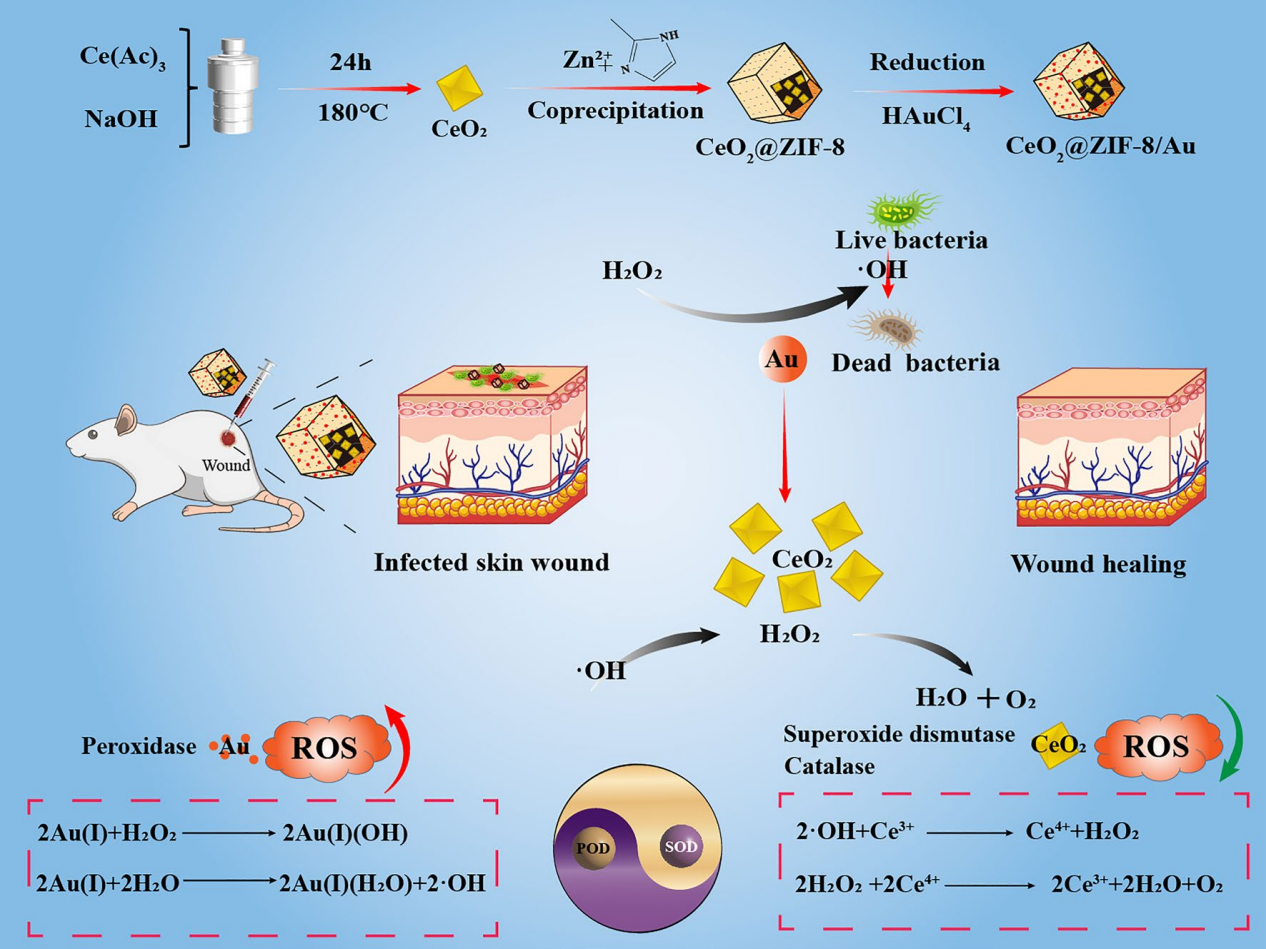
CeO_2_@ZIF-8/Au NPs treatment and healing promoting mechanism of bacterial infected wound in mice and equations of ROS production/clearance

Herein, we have developed a core–shell nanozyme (CZA) that spontaneously active both ROS generating and scavenging functions, balancing ROS steady-state to promote wound healing. ZIF-8, as a metal–organic framework (MOF), has non-toxic and good biocompatibility, and mainly acts as a bridge between two different functional components. Firstly, The Au NPs on the shell of the ZIF-8 material rapidly exhibit its POD-like activity, utilizing the characteristics of high-efficiency of producing ROS to kill bacteria. Meanwhile, CeO_2_ core is encapsulated in ZIF-8, temporarily limiting its SOD and cat-like activity. Subsequently, as the ZIF-8 structure gradually decomposes in the acidic microenvironment, the CeO_2_ core is gradually released, exerting its SOD and CAT-like activity to eliminate excess ROS produced by the Au NPs. These two functions automatically and continuously regulate the balance of ROS levels, ultimately achieving the function of killing bacteria, reducing inflammation, and promoting wound healing (Fig. 1).

## Experimental Section

### Materials

Cerium acetate (≥ 99%, (CH_3_COO)_3_Ce), 4′,6-diaminidine-2-phenylindole and propidium iodide bought in Jiangsu KeyGEN BioTECH Corp. 5,5-dimethyl-1-pyrrolin-*N*-oxide was bought in Shanghai Aladdin Biochemical Technology Co. Zinc nitrate hexahydrate (≥ 99.7%), chloroauric acid tetrahydrate (≥ 47.8%), sodium borohydride (96%), sodium chloride (99.7%) were purchased from Sinophinacea Chemical Reagents Co., LTD. Polyvinylpyrrolidone (PVP; Mw = 3500) was purchased from Aladdin. *N*,*N*-dimethylformamide (DMF), hydrogen peroxide (30%, H_2_O_2_), sodium hydroxide, hydrochloric acid and absolute ethanol (≥ 99.7%, C_2_H_6_O) were purchased from Xilong Technology Co., LTD. LB Broth, which contains peptone, yeast extract, and NaCl, was procured from Sangon Biotech. LB broth agar, which contains peptone, yeast extract, agar, and sodium chloride, was bought in Dalian Meilun Biotechnology Co. 2-methylimidazole was bought in Onokai Technology Co. Solutions are formulated via ultrapure H_2_O (18.2 MΩ cm).

### Characterization

X-ray diffraction (XRD) were tested on Bruker SmartLab SE X-ray powder diffractometer. UV–vis spectra were tested on Shimadzu Cary 5000 UV–vis spectrophotometer. Transmission electron microscopy (TEM) photographs were acquired on JEM-1400, while elemental data obtained using Tecnai F30. Scanning electron microscopy (SEM) photographs were characterized using ZEISS Sigma SEM at 15 kV. Hydrodynamic diameter and ζ potential were determined via DLS on Malvern ZEN 3600. Reactive oxygen species was measured with ESR (EMX Bruker-10/12). Fluorescence imaging was recorded using a Leica DM 6000 B, and bacterial counts and area calculations were performed using NIH ImageJ software.

### Preparation and Characterization of CeO_2_ Nanoparticles

CeO_2_ nanoparticles were synthesized using the solvothermal method. Initially, 0.1 M Ce(Ac)_3_ and 0.02 M NaOH were dissolved in 80 mL of ultrapure H_2_O, stirred until completely homogenized. The resultant mixture was then transferred to a high-pressure reaction vessel. The reaction was carried out at 180 °C for 24 h. The obtained product was purified 3 times using ultrapure H_2_O at 12,000 rpm and subsequently dried under vacuum to obtain CeO_2_ nanoparticles.

### Preparation of CeO_2_@ZIF-8 Nanoparticles

CeO_2_ nanoparticles (35 mg) and PVP (1 g) were separately dissolved in methanol (50 mL), and then mixed for 24 h at 25 °C. The product was centrifuged and redispersed in 50 mL of 25.6 mM 2-methylimidazole (in methanol solvent) immediately, and then was stirred for 20 min at 25 °C until it was thoroughly mixed. Subsequently, we added Zn (NO_3_)_2_·6H_2_O (50 mL of 25.2 mM in methanol solvent) and mixed for an additional 30 min until complete reaction occurred to yield the product. The final product was centrifuged at 12,000 rpm, purified 3 times with methanol, and vacuum-dried.

### Preparation of CeO_2_@ZIF-8/Au Nanoparticles

To synthesize CZA nanoparticles, we employed the reduction method. Firstly, 50 mg of prepared CZ NPs were dispersed evenly in methanol (50 mL) by sonication for 20 min. Next, added 150 μL of 0.1 M HAuCl_4_·3H_2_O and ultrasonic mixed for 20 min, then moved to a magnetic stirring device and stirred at 800 rpm. During the stirring process, quickly added freshly prepared NaBH_4_ solution (0.1 M, 3.75 mL), and the stirring speed of 800 rpm was maintained for another hour. The samples were centrifuged at 6000 rpm, purified 3 times with methanol, and vacuum-dried for 12 h to obtain the final product, CZA nanoparticles.

### Hydroxyl Radical Assay and ESR Assay for Free Radical Scavenging

DMPO acts as a free radical catcher that can trap hydroxyl radicals and form adducts that can be detected by an ESR spectrometer. In the experiment, CZA (200 μg mL^−1^), DMPO (50 mM) and of H_2_O_2_ (5 mM) were thoroughly mixed. After 10 min of reaction, ESR signal was detected and recorded. ·OH in the free radical scavenging experiment was generated from FeCl_2_ (1.8 mM) and H_2_O_2_ (5 mM). The ESR signal was detected and recorded by adding 200 μg mL^−1^ of CZA in PBS buffer (pH = 4.5) for 4–24 h at 25 °C and reacting for 30 min.

### Catalytic Analysis

In this experiment, the catalytic activity of the materials was evaluated using 3,3′,5,5′-tetramethylbenzidine (TMB) as an indicator. The colorimetric reaction was performed in a quartz cuvette using a mixture containing 500 μmol mL^−1^ H_2_O_2_, 3.2 mM TMB, NaAc buffer solution, and 200 μg mL^−1^ of the material (CeO_2_, CZ, and CZA). After the color stabilized, the UV absorption spectra of the solution were measured and recorded in the range of 350–800 nm.

TMB was also used as the indicator, and the steady-state kinetics of the material was analyzed by UV–vis spectrophotometer in a quartz cuvette. Firstly, the impacts of different pH values on the catalytic property of ZIF-8/Au were studied. Prepare 20 mM NaAc buffer, then adjust its pH with hydrochloric acid, and prepare NaAc buffer with pH = 3.5, 4.5, 5.5, 6.5 for use. Add 3.2 mM TMB, CZA and NaAc buffer to the UV dish, stir evenly, and place in the UV detection device. Finally add H_2_O_2_, stir quickly, and detect immediately at 652 nm in UV–vis spectrophotometry. The change curves of ultraviolet absorption intensity of the system were recorded within 0–10 min. Set the pH of NaAc buffer to 4.5 and the H_2_O_2_ concentration of 5, 12.5, 25, and 50 mM, respectively. We used Eqs. (1–3) as below:

1 $$A=kbc$$

2 $$ V_{0} = (V_{\max } \cdot\left[ S \right]) / (K_{m} + \left[ D \right]) $$

3 $$ 1/V_{0} = K_{m} / (V_{\max } \cdot\left[ S \right]) + 1/V_{\max } $$

Here *A* is absorbance value, and *K* represent the molar extinction coefficient, *b* is the thickness of the absorption layer, *c* is substrate’s concentration. Here, *k* = 39,000 M cm^−1^, and *b* = 1 cm.

### In Vitro Antibacterial Assays

In the context of antimicrobial research, *E. coli* and *S. aureus* were selected as representative Gram-negative and Gram-positive bacteria, respectively. We procured Strains of *E. coli* (ATCC25922) and *S. aureus* (ATCC29213) in Xiang'an Hospital. Bacterial culture medium utilized LB broth of 25 g L^−1^ and pH of 4.5. In addition, the solid agar medium was constructed using ultrapure water prepared with agar powder involving LB broth (0.04 g mL^−1^). MIC and MBC against *E. coli* were determined with or without H_2_O_2._ Various concentrations of CZA (15, 30, 60, 120, 240 μg mL^−1^), 100 μmol L^−1^ of H_2_O_2_ and 10^6^ CFU mL^−1^ of *E. coli* were blended in the above broth solution and maintained at 37 °C overnight. MIC against *E. coli* was determined by microplate reader at 595 nm. After diluting bacterial suspension 10^6^-fold, which incubated on agar plates for sixteen hours, counted number of bacterial colonies, and established MBC against *E. coli.* Additionally, the material concentration for Staphylococcus aureus was adjusted to 1.25, 2.5, 5, 10, and 20 μg mL^−1^.

Then 10^6^ CFU/mL of *E. coli* were treated with normal saline, H_2_O_2_, CeO_2_, CZ, CZA, CZA + H_2_O_2_ (the concentrations of CeO_2_, CZ, and CZA were 120 μg mL^−1^, respectively, and the concentration of H_2_O_2_ was 100 μmol L^−1^). The bacterial suspension was maintained at 37 °C for 16 h and subsequently diluted 10^6^-fold. The diluted suspension was then cultured on agar plates for another 16 h to facilitate the enumeration of bacterial colonies. Similarly, Staphylococcus aureus was handled in the same manner, and the concentrations of CeO_2_, CZ, and CZA were adjusted to 10 μg mL^−1^.

### SEM Morphology of Bacterial Specimens

Bacterial cells collected via centrifugation (5000 rpm, 5 min) and purified 2–3 times with PBS (pH = 7.4). Next, soaked in 2.5% glutaraldehyde and kept at 4 °C for 1 h. The fixed specimens were then progressively dehydrated with an increasing concentration of ethanol solution (10%, 30%, 50%, 70%, and 90%) for ten minutes. In the end, the specimens were dispersal in absolute ethanol.

#### TEM of Bacterial Specimens

Bacterial cells collected via centrifugation (5000 rpm, 5 min) and purified 2–3 times with PBS (pH = 7.4). Next, soaked in 2.5% glutaraldehyde and kept at 4° for 1 h. The bacterial specimens were then embedded in agar, sectioned with an ultrathin microtome and observed by TEM.

#### Live/Dead Bacteria Stain

Bacterial cells centrifuged and purified via sterile PBS. Subsequently, the treated bacteria were stained with 1.5 μmol L^−1^ of 4',6-diaminine-2-phenylindole (DAPI) and 1.6 μmol L^−1^ of propidium iodide (PI) for ten minutes. Purified bacteria via sterilized PBS to remove any free dye and surveyed through polarizing fluorescence microscope.

### Protein Leakage

Purified bacterial cells with sterilized PBS. Collected the supernatant by centrifugation at 4 °C and added into 96-well plate. Then measured the protein concentration at 595 nm using the BCA Protein Detection Kit and Microplate Reader.

### Cell Culture

L929 cells and 293T cells were grown in MEM (contain non-essential amino acid, PM150410, Pricella) or DMEM (SH30243.01, HyClone) medium containing 10% FBS (FBS500-A, HYCEZMBIO), penicillin streptomycin solution (1:100, SXQ006, HyClone), respectively. The cells were incubated in a humidified incubator at 37 °C with 5% CO_2_. Subcultures of cells were performed at 80–90% confluence using 0.25% trypsin–EDTA (SH30042.01, HyClone). CZA was immersed in PBS (pH 4–4.5) for 2 h (CZA-2 h) or 8 h (CZA-8 h), respectively, to degrade the ZIF-8 structure in the acidic microenvironment. 200 μM H_2_O_2_ and 2 μg mL^−1^ LPS (Sigma-Aldrich, L2880) was applied to damage the L929 cells. Materials CeO_2_, CZ, CZA, CZA-2 h, CZA-8 h with a concentration of 80 ug mL^−1^ were added to the cell culture medium.

### Cytotoxicity Assay

L929 cells or 293T cells were carefully seeded in a 96-well plate at a density of 5 × 10^5^ cells per well and allowed to grow for 24 h, respectively. 30 and 60 ug mL^−1^ CZA were incubated in cell culture medium. After 12, 24, or 48 h incubation, the cells viabilities were investigated through a Cell Counting Kit-8 assay (G4103-1ML, Servicebio). Cell apoptosis was further analyzed by Calcein-AM cell apoptosis assay (G1609-100T, Servicebio). After 50 μg mL^−1^ CZA were incubated in cell culture medium for 24 h, the cells in each group were suspend using 0.25% trypsin–EDTA. Then the cells were incubated in Calcein-AM and PI for 30 min and were observed with a fluorescence microscope at 490 and 545 nm laser to identify the live and dead cells. Take 4 views from each culture dish for observation, with a total of 12 views from each group. Count and count the number of dead and surviving cells in each field separately. ImageJ software is used to count cell numbers. After cells in each group were fixed with 4% paraformaldehyde (4 ℃, 15 min), phalloidin (G1041, Servicebio) was used to stain the cytoskeleton of cells, and DAPI (G1012, Servicebio) was used to stain the nucleus of cells. Confocal microscopic images are used to observe the cytoskeleton.

### ROS Assays

After adding various materials to the cell culture medium for 24 h, the cell were washed by PBS buffer remove excess material. 200 μM H_2_O_2_ was applied to L929 cells for 1 h. After washing the cells again with PBS buffer, DCFH-DA fluorescent probe (S0033M, Boyetime) was utilized to detect the endogenous ROS levels of L929 cells. Finally, the cells were observed with a fluorescence microscope.

### Real-Time PCR

Total RNA was extracted from L929 cells or 293T cells with ExTrizol Reagent (Protein Biotechnology, PR90) and reverse transcribed to cDNA by using PrimeScript™ RT reagent Kit with gDNA Eraser (RR047A, Takara). The qRT-PCR was performed on a LightCycler 480 RT-PCR system (Roche Diagnostics Ltd, Switzerland) with the TB Green® Premix Ex Taq™ (RR420A, Takara). The following primers were designed for each targeted mRNA or DNA:

L929 cell primers:

- SOD1 (F): 5′- CACTCTAAGAAACATGGTGG-3′,-
- SOD1 (R): 5′- GATCACACGATCTTCAATGG-3′,
- SOD2 (F): 5′- CTTCAATAAGGAGCAAGGTC-3′,
- SOD2 (R): 5′- CAGGTCTGACGTTTTTATACTG-3′,
- GAPDH (F): 5′- GGGAAGCCCATCACCATCTTC-3′,
- GAPDH (R): 5′- AGAGGGGCCATCCACAGTCT-3′,
- Caspase-3 (F): 5′- AAGATACCGGTGGAGGCTGA -3′,
- Caspase-3 (R): 5′-AAGGGACTGGATGAACCACG‐3′,

293T cell primers:

- SOD1 (F): 5′-TGGTTTGCGTCGTAGTCTCC‐3′,
- SOD1 (R): 5′-CTTCGTCGCCATAACTCGCT‐3′,
- SOD2 (F): 5′-CAGGCAGCTGGCTCCGGTTT‐3′,
- SOD2 (R): 5′-TGCAGTGGATCCTGATTTGG‐3′,
- GAPDH (F): 5′- GCGAGATCCCTCCAAAATCAA-3′,
- GAPDH (R): 5′- GTTCACACCCATGACGAACAT-3′,
- Caspase-3 (F): 5′- GGAAGCGAATCAATGGACTCTGG-3′,
- Caspase-3 (R): 5′- GCATCGACATCTGTACCAGACC-3′,
- IL-6(F): ACTCACCTCTTCAGAACGAATTG,
- IL-6(R): CCATCTTTGGAAGGTTCAGGTTG,
- TNF-α(F): CCTCTCTCTAATCAGCCCTCTG,
- TNF-α(R): GAGGACCTGGGAGTAGATGAG,

qPCR conditions consisted of an initial denaturing step of 5 min at 95 °C followed by 45 cycles of 10 s denaturation at 95 °C, 20 s annealing at 60 °C, and 20 s extension at 72 °C. The mRNA expression was normalized to the mRNA expression of GAPDH. The results were calculated using the comparative cycle threshold (ΔΔCt) method.

### Cell Migration

The rate of cell migration plays an important role in wound healing, so in vitro scratch assays were performed to investigate the effect of synthetic materials on cell migration activity. A certain density of cell suspensions was seeded into 24-well plates, and the criterion was that cells could cover more than 90% of the well area after 24 h. After 24 h of culture, each well was scraped vertically with a 200 μL sterile pipette tip and washed three times with PBS to remove unattached cells. HUVECs cells were then cultured with DMEM containing CeO_2_, ZIF-8, CZ and CZA NPs with 5% FBS at a concentration of 80 μg mL^−1^ of the sample in each well. Cells cultured in the absence of samples were treated as controls. The purpose of adding 5% FBS to DMEM was to slow cell proliferation while maintaining cell survival to eliminate interference. After 24 h of incubation, the morphology of HUVECs cells was observed under an inverted fluorescence microscope. Migration assays were performed using imageJ software to quantitatively measure the area of the initial scratch (*S*_0_) and healing scratch (*S*_*t*_). Mobility was calculated as Eq. (4):

4 $${\text{mobility}} = (1-S{\text{t}}/S0) \times 100\%$$

### Anti-infection Experiment In Vivo

All BALB/c mice used in the experiments were obtained from Beijing Vitarui Animal Technology Co., LTD and were 6–8 weeks old. All experiments complied with the rules in the Animal Central of Xiamen University. Mice were injected with 200 μL *S. aureus* containing 10^7^ CFU mL^−1^. After 24 h of incubation of the bacteria subcutaneously, 4% chloral hydrate was used to anesthetize mice and a 7 mm diameter skin wound was cut at the injection site. Then those mice were stochasticly assigned into 7 groups. The above groups were set as one control group and six experimental groups. Among them, control set conducted with PBS (10 mM, 20 μL), meanwhile, 6 experimental groups were separately treated with 20 μL H_2_O_2_ (4 mM), 20 μL CeO_2_ (100 μg mL^−1^), 20 μL CZ (100 μg mL^−1^), 20 μL CeO_2_@ZIF-8/Au (100 μg mL^−1^), 10 μL H_2_O_2_, ZIF-8/Au (200 μg mL^−1^) + 8 μL H_2_O_2_ and 10 μL CeO_2_@ZIF-8/Au (200 μg mL^−1^) + 8 μL H_2_O_2_. The drug was given to cover the wounds of mice. The treatment day was set at day 0, and body weight and wound size were recorded every other day for all groups of mice. ImageJ software was used to measure the wound area. After 7 days, mice were sacrificed, and wounds, hearts, liver, spleen, lungs and kidneys were taken for H&E staining analysis.

## Results and Discussion

### Preparation and Characterization of CeO_2_@ZIF-8/Au Nanoparticles

**Fig. 2:**
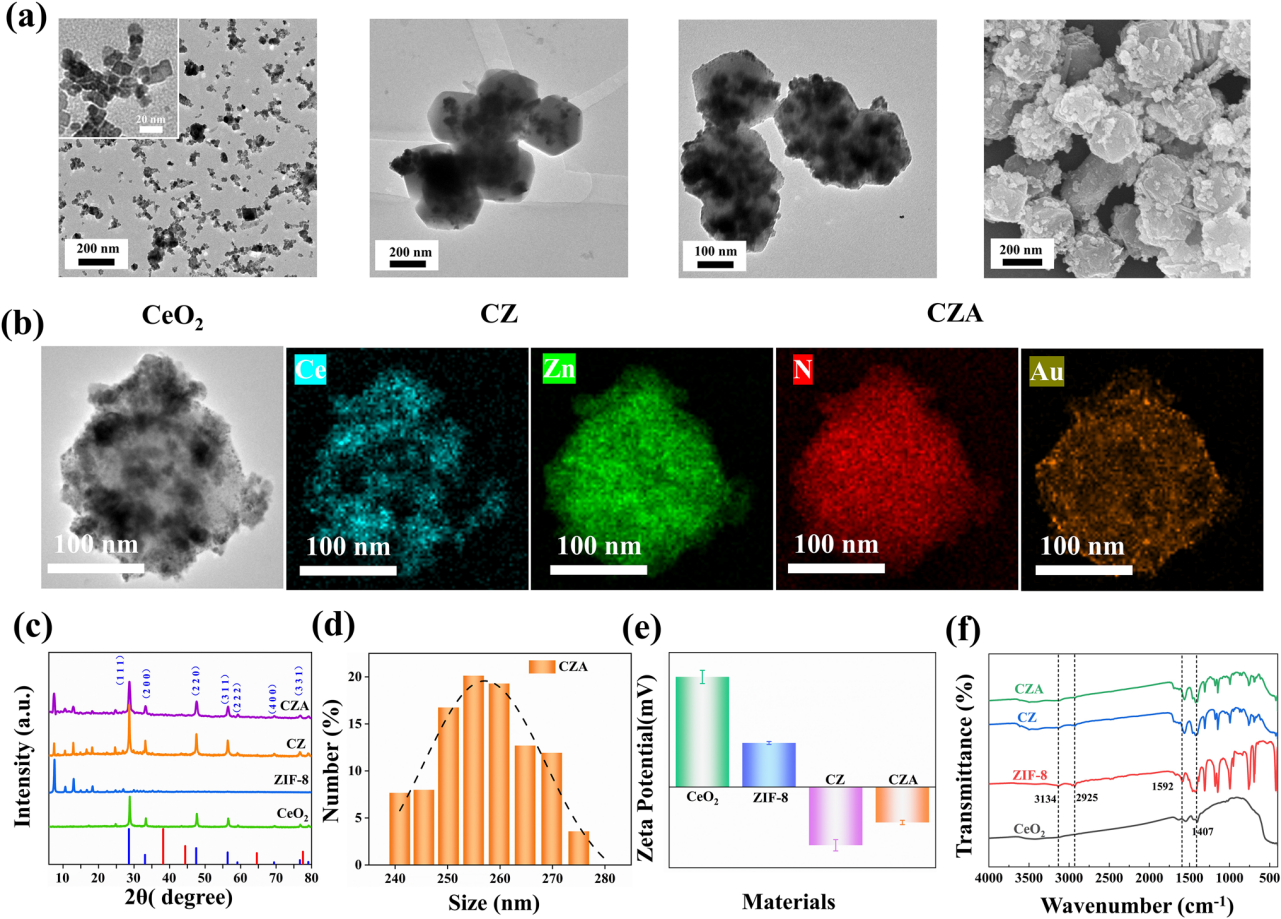
Characterization of basic material properties. **a** TEM images of CeO_2_, CZ and CZA. SEM images of CZA. **b** High resolution image of CZA and EDS elemental mapping images (Ce, Zn, N, Au). **c** XRD of CeO_2_, ZIF-8, CZ and CZA. **d** Hydrodynamic diameter of CZA. **e** ζ-Potentials of CeO_2_, ZIF-8, CZ, and CZA (Dispersed in methanol, pH = 7.0). **f** FT-IR spectra of CeO_2_, ZIF-8, CZ and CZA

Firstly, CeO_2_ nanoparticles were synthesized by solvothermal method. ZIF-8 was then allowed to grow on the outside of CeO_2_ nanoparticles to form CeO_2_@ZIF-8 (CZ). Finally, HAuCl_4_ was reduced by the original reduction method so that the Au nanoparticles were loaded on the surface of CZ. TEM revealed that the synthesized CeO_2_@ZIF-8/Au (CZA) nanoparticles presented rhomboid dodecahedral structure. The mean diameter is 150 ± 25 nm. CeO_2_ nanoparticles presented a cubic structure, and the average diameter is 10 ± 5 nm (Fig. 2a). From Fig. 2a, it can be seen that CeO_2_ nanoparticles are successfully wrapped in ZIF-8, and Au nanoparticles can be observed uniformly distributed on the surface of CZA after being loaded on CZ. At the same time, this process does not change the shape of nanoparticles. However, due to the acid etching of HAuCl_4_, the particle size of CZA is smaller than that of CZ. SEM images of CZA can also clearly observe its rhombohedral dodecahedron shape decorated with gold nanoparticles, which are consistent with TEM images. Furthermore, Fig. 2b, which shows the EDS data, illustrates the distribution of Ce, Zn, N, and Au throughout the entire CZA architecture. The presence of Ce can be attributed to CeO_2_, while Zn and N can be attributed to ZIF-8. Additionally, the presence of elemental Au is likely due to the gold nanoparticles.

The X-ray diffraction (XRD) peaks of CZA is almost identical to that of CZ (Fig. 2c), and the position of the characteristic peak can exactly correspond to that of the standard card of CeO_2_ and the characteristic peak of ZIF-8, which suggests that the crystal structure of CZA remains unchanged compared to that of ZIF-8, and the crystal structure does not change during the loading of gold nanoparticles on the surface. However, in the XRD patterns of CZA, the characteristic peaks corresponding to the gold nanoparticles could not be found, which may be due to the fact that the gold nanoparticles are too small (~ 5 nm) to be detected. Dynamic light scattering analysis revealed that the mean size of hydrated particles in CZA was 250 ± 20 nm, as depicted in Fig. 2d. Through Zeta potential analysis, the ζ-Potentials of CeO_2_, ZIF-8, CZ, and CZA are + 43.67, + 15.20, − 23.97, and − 13.65 mV, respectively (Fig. 2e). Be able to tell that the formation of CZ changes the ζ-Potentials magnitude of CeO_2_ or ZIF-8. This phenomenon is similar to the literature [8], but the exact reason is unclear yet. Notably, the positively charged gold nanoparticle loading makes the ζ potential of CZA higher than that of CZ. Zeta potential analysis further confirmed the successful synthesis of CZA material. Figure 2f shows the Fourier transform infrared spectroscopy (FT-IR) spectra of each component of the material ranging from 4000 to 400 cm^−1^. The peaks appearing around 1407 and 1557 cm^−1^ are the stretching vibration peaks of Ce–O bond, and the peaks around 1645 cm^−1^ are the bending vibration peaks of H–O–H bond. The absorption peaks of 3134, 2925, and 1592 cm^−1^ in the infrared spectra of ZIF-8 are caused by the stretching vibration of aromatic C–H bond, the stretching vibration of fat C–H bond and the stretching vibration of C–N bond in 2-methylimidazole, respectively. The absorption peak at 420 cm^−1^ is the stretching vibration peak of the Zn–N bond, which is the bond formed by the coordination of Zn^2+^ and 2-methylimidazole. Among them, the absorption peak at 1592 cm^−1^ is slightly redshifted in the infrared spectra of CZ and CZA, and the characteristic absorption peak of CeO_2_ at 1407 cm^−1^ can also be observed in the infrared spectra of CZ and CZA. In short, the infrared spectra also indicate the successful synthesis of above materials.

### Catalytic Performance of CeO_2_@ZIF-8/Au and ROS Scavenging

**Fig. 3:**
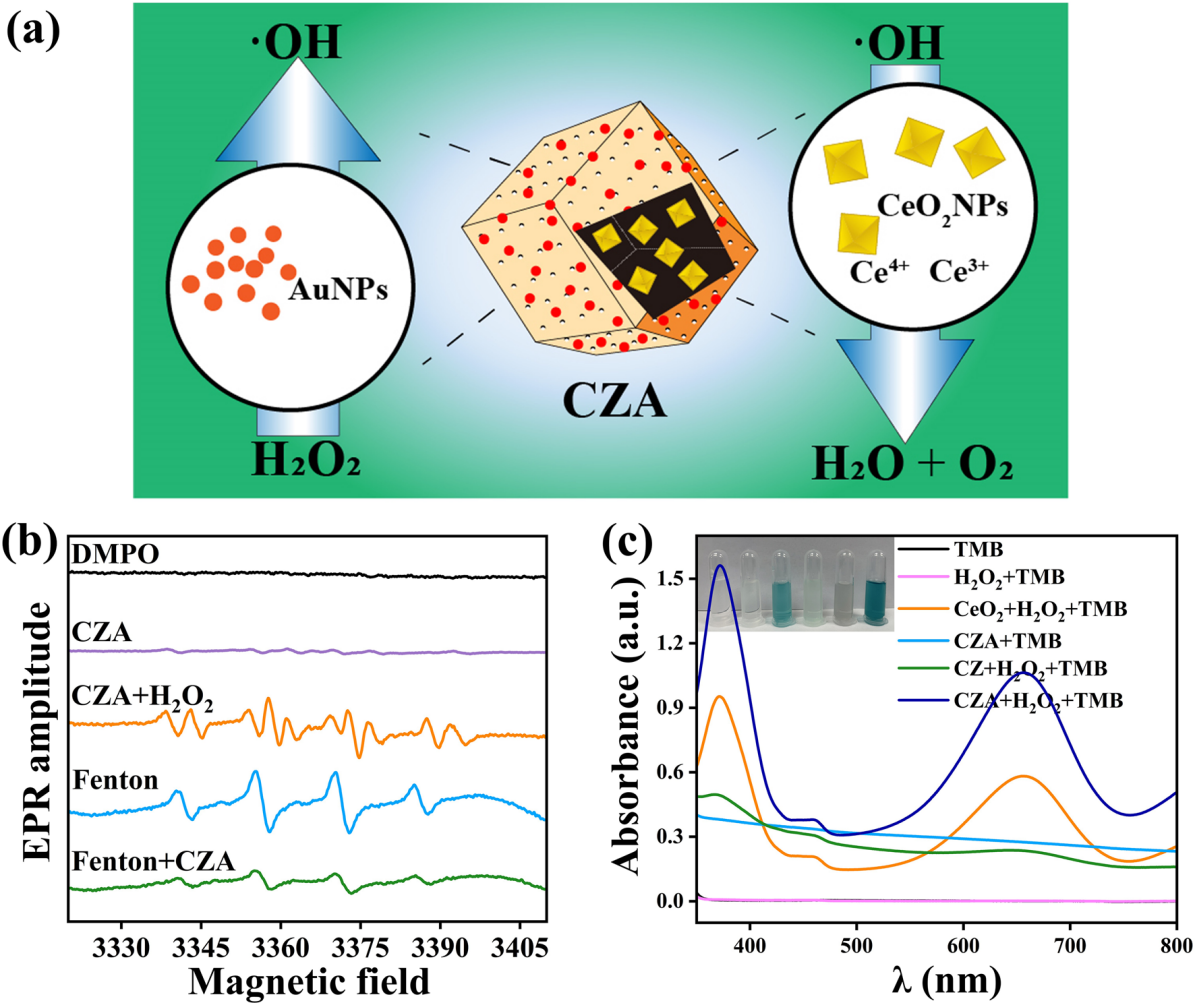
Characterization of materials catalytic properties. **a** schematic diagram of the corresponding catalytic action of the inner (CeO_2_ NPs) and outer layers (Au NPs) of CZA. **b** ESR spectral of various groups. **c** UV–vis absorption of TMB in various groups (pH 4.5): TMB, H_2_O_2_ + TMB, CeO_2_ + H_2_O_2_ + TMB, CZA + TMB, CZ + H_2_O_2_ + TMB, CZA + H_2_O_2_ + TMB

Figure 3a is a schematic diagram of the corresponding catalytic action of the inner (CeO_2_ NPs) and outer layers (Au NPs) of CZA. After the successful preparation of CZA, electron spin resonance spectroscopy was utilized to explore the ability of CZA to catalyze free radical production and free radical scavenging. To trap reactive oxygen species (ROS), 5,5-Dimethyl-1-Pyrroline *N*-oxide (DMPO) was used as a capture agent. The electron spin resonance test detected characteristic signals of the 1:2:2:1 ratio, confirming that the ROS generated by the CZA catalytic reaction are highly active and toxic hydroxyl radicals (·OH), as shown in Fig. 3b. This result ensures the effective bactericidal performance of CZA (Fig. 3b). The characteristic peak of ·OH produced by CZA catalysis shows some miscellaneous peaks, which may be due to the influence of organic components in ZIF-8 on the detection. In the fenton reaction group, the peak intensity of ·OH which produced by standard fenton reaction was high. However, after CZA (acid hydrolysis for 12 h at pH = 4.5 buffer) was added (Fenton + CZA group), the peak intensity of ·OH decreases significantly. This indicates that after acid immersion, the ZIF-8 framework of CZA is broken and inner CeO_2_ NPs were released, played the role of free radical scavenging.

Meanwhile, the catalytic performance of CZA was studied by UV–vis spectrophotometer with 3,3',5,5'-tetramethylbenzidine (TMB) as indicator. Figure 3c shows that only the CeO_2_ + H_2_O_2_ and CZA + H_2_O_2_ groups exhibited color reaction, with characteristic absorption peaks appearing in the UV–visible characteristic absorption peaks at 370 and 652 nm. CeO_2_ has suitable oxidation vacancies and the ability to rapidly convert Ce ions between tetravalent and trivalent ions. Thus, in acidic environments, CeO_2_ can exert peroxidase activity. POD activity of CeO_2_ catalyzes hydrogen peroxide to product · OH, which reacts with TMB to form oxTMB with two absorption peaks at 370 and 652 nm. Conversely, in Fig. 3c, the CZ + H_2_O_2_ group did not reveal characteristic absorption peaks at 370 and 652 nm, which indicated that CZ did not exhibit any simulated enzyme activity, further demonstrating the encapsulation of CeO_2_ in ZIF-8 frame structure can restrict the active site indicated by CeO_2_ and limit the catalytic performance of CZ. In addition, the absorbance intensity of CZA + H_2_O_2_ groups at 370 and 652 nm is the highest, indicate that the catalytic performance of Au nanoparticles is stronger than that of CeO_2_ nanoparticles.

**Fig. 4:**
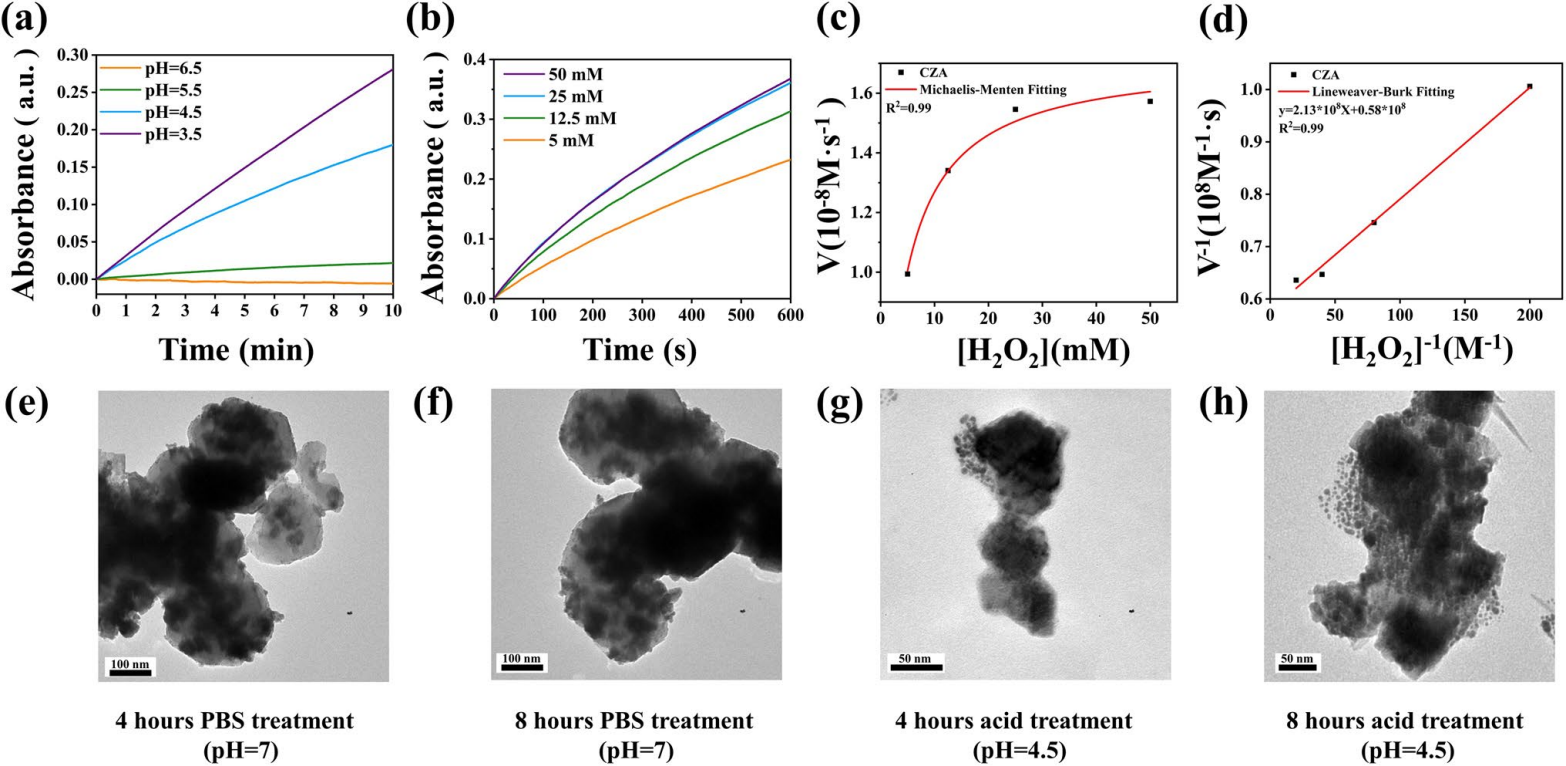
Characterization of catalytic kinetics and acid degradation ability of materials **a** The absorbance value of TMB chromogram of CZA (100 μg mL^−1^) at varied pH values (3.5, 4.5, 5.5, and 6.5) when 5 mM H_2_O_2_ was added. Time-course absorbance of **b** Time-course absorbance of CZA with different H_2_O_2_ (50, 25, 12.5, and 5 mM) and Michaelis–Menten **c** kinetics and Lineweaver–Burk **d** curves of CZA. TEM images of CZA **e, f** after PBS treatment (pH = 7) for 4 and 8 h, **g, h** after acid treatment (pH = 4.5) for 4 and 8 h

Due to the significant impact of pH on the peroxidase-like activity of nanozymes, we investigated the influence of pH on the CZA catalytic performance, as illustrated in Fig. 4a. The data reveals that the catalytic performance of CZA gradually improves with decreasing pH, with significant enhancement observed when pH < 5. Therefore, in the subsequent experiments, the catalyzed reactions involving CZA were all carried out in the pH = 4.5 system.

Typical Mie steady-state kinetics was used to evaluate catalytic capacity of CZA. Figure 4b shows the change of UV absorbance intensity of CZA with time when the concentration of substrate H_2_O_2_ increases gradually. Based on the date in Fig. 4b, according to different substrate concentration and the calculated initial velocities (*V*_0_), the Michaelis–Menten equation was used to fit, and the fitting curve was drawn (Fig. 4c). Meanwhile, Fig. 4d was drawn using Mie equations based on Fig. 4c. As shown in Fig. 4c, d and based on the Mie equations, the Michaelis–Menten constant (*K*_m_) and the maximum velocity (*V*_max_) were 3.69 mmol L^−1^ and 1.73 × 10^–8^ M s^–1^.

In addition, we further studied the structural changes of CZA at different time in different pH environments by TEM. As is shown in Fig. 4e, f, after PBS treatment (pH = 7) for 4 and 8 h, no morphological changes occurred for CZA NPs. While after acid treatment (pH = 4.5) of CZA for 4 and 8 h, part of the structure of CZA gradually collapsed and released small nanoparticles with the average diameter of 10 ± 5 nm, which is corresponded with the size of CeO_2_ nanoparticles. This demonstrate that the ZIF-8 frame structure decomposed in the acidic microenvironment and released CeO_2_ nanoparticles from the core, which confirmed the design of automatically and continuously regulate the balance of ROS levels with CZA nanoplatform.

### Evaluation of Antimicrobial Properties In Vitro

**Fig. 5:**
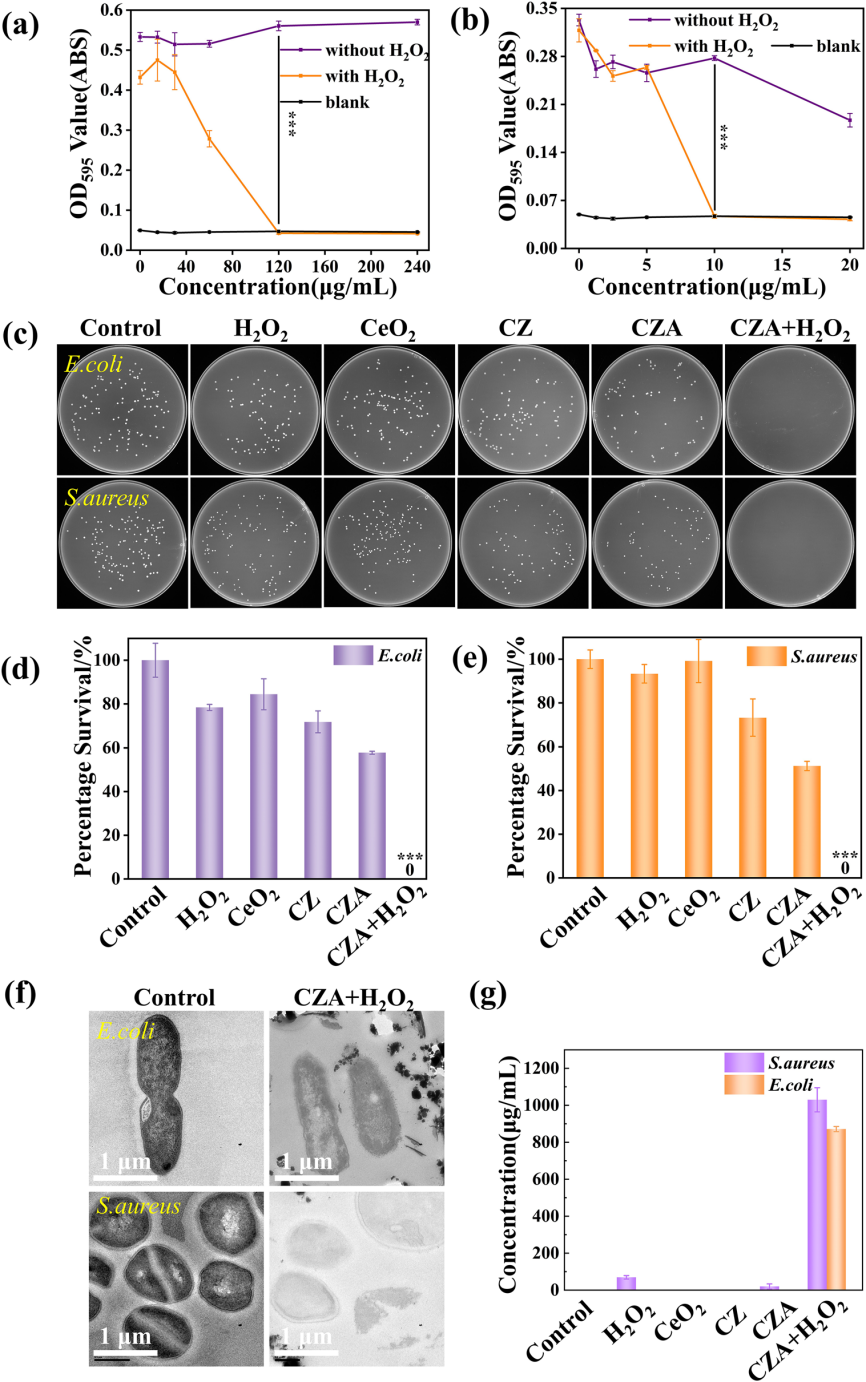
Characterization of antibacterial activity of materials in vitro. MIC of CZA NPs incubated with **a** *E. coli* and **b** *S. aureus*. **c** Agar plates photographs with remaining inoculated bacteria (above, *E. coli*; lower, *S.aureus*). **d, e** Bar chart of bacterial survival numbers. **f** TEM photographs of *E. coli* and *S. aureus*. **g** Protein exudation of bacterial in various group (mean ± SD, *n* = 3; *P* values: *P < 0.05, **P < 0.01, ***P < 0.001)

Due to the catalytic properties of CZA, we used Gram-negative strain *E. coli* and Gram-positive strain *S. aureus* to study the antibacterial performance. In a typical in vitro antimicrobial assay, both bacteria were cultured with a gradient increase in concentrations of CZA, while substrate H_2_O_2_ was provided at a concentration of 100 μmol L^−1^ in one of them to trigger the CZA catalytic reaction. After 16 h of incubation, tested the absorbance of the bacterial mixture at 595 nm using a microplate reader (OD_595_). The miscible liquids aforementioned were diluted at 10^6^-fold and inoculated on solid LB agar plates to appraise bactericidal performance. The results depicted in Figs. 5a, b and S2 indicate a significant decrease in absorbance of both bacterial strains at OD_595_ with the increase in CZA concentration in the H_2_O_2_ group. The concentration in Fig. 5a is 120 μg mL^−1^, and the concentration in Fig. 5b is 10 μg mL^−1^. Finally, the OD_595_ curves of both bacteria in H_2_O_2_ group contacted with the OD_595_ curves of blank group (LB culture medium) at CZA concentrations of 120 and 10 μg mL^−1^, respectively. At the same time, the culture medium of the system showed a clear state at this concentration. The aforementioned concentration, 120 and 10 μg mL^−1^ for *E. coli* and *S. aureus*, respectively, represents the minimum inhibitory concentration (MIC) of CZA against these two bacterial strains. Minimum bactericidal concentration (MBC) were further colony counting on agar plates. The results (Fig. S2) showed that *E. coli* survival with 120 μg mL^−1^ CZA under H_2_O_2_ treatment was 0. Similarly, *S. aureus* survival with 10 μg mL^−1^ CZA was also 0, which is consistent with the findings shown in Fig. 5a, b. The above results indicate that, the MBC of CZA for *E. coli* was 120 and 10 μg mL^−1^ for *S. aureus*. In order to evaluate the antibacterial effects of CZA, we treated *E. coli* and *S. aureus* with PBS (control), H_2_O_2_, CeO_2_, CZ, CZA, and CZA + H_2_O_2_ at concentrations of 120 and 10 μg mL^−1^, respectively. Incubated overnight, the culture medium was diluted and cultured on agar plates. The results are presented in Fig. 5c. We also conducted statistical analysis of the bacterial colonies in each group and presented the results in Fig. 5d, e. The findings show that *E. coli* and *S. aureus* with CZA + H_2_O_2_ did not survive, while the bacterial growth in other groups was only weakly affected compared to the control group. Moreover, a cross-sectional index of bacterial membrane damage is protein leakage. Therefore, we detected the protein concentration using protein detection kits for bacteria treated with different groups (Fig. 5g). The protein leakage concentration was found to be the highest only in the bacteria treated with the CZA + H_2_O_2_ group (871 μg mL^−1^ for *E. coli*, 1029 μg mL^−1^ for *S. aureus*) and other groups did not detect protein leakage, indicating CZA can damage cell membranes in the presence of H_2_O_2_ and further confirming the antibacterial effect of CZA.

**Fig. 6:**
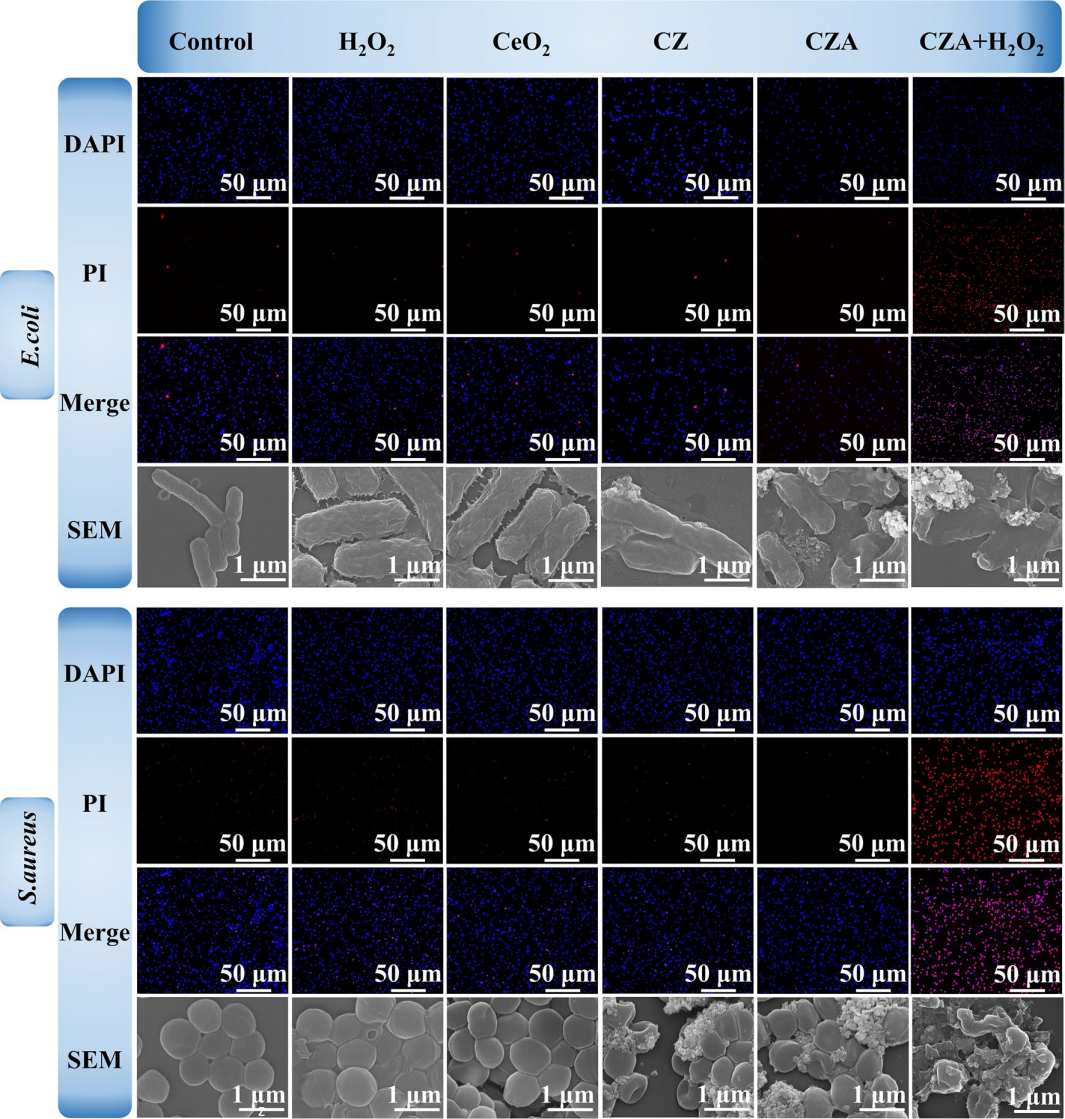
Live/Dead staining confocal microscopy and SEM photographs of *E.coli* and *S.aureus* after different treatments

SEM (Fig. 6) and TEM (Fig. 5f) images were used to observe the morphology of bacteria treated by different groups. Observations reveal that in the bacteria treated with CZA + H_2_O_2_, the bacterial membranes appeared broken or sunken and were clearly visible. Additionally, the leaked cell contents caused some of the bacteria to stick to each other, resulting in incomplete bacterial structures. This phenomenon is attributed to the catalytic properties of CZA, which can convert the substrate H_2_O_2_ into toxic ·OH. Even at low dosages, the resulting ·OH is capable of directly damaging the bacterial membranes, divulgating cell contents. As such, CZA has a high potential for eradicating bacteria in various biological applications.

To demonstrate the antibacterial properties of CZA more visually, we used DAPI and PI to stain all bacteria, and observed with polarized fluorescence microscope. DAPI could cross the cell walls and membranes of bacteria, and bind to the DNA inside the bacteria. PI cannot cross the cell membrane of bacteria, but can only enter the bacteria through the broken cell membrane, embedded in DNA, so it can only cause dead bacteria to emit red fluorescence. As can be seen from Fig. 6, for the two representative bacteria, only the CZA + H_2_O_2_ group showed strong red fluorescence, which means that in the presence of substrate H_2_O_2_, CZA could exert a broad spectrum of bactericidal activity. Meanwhile, the other groups only showed strong blue fluorescence, indicating that the bacteria treated by the other treatment groups did not have too much survival pressure.

### In Vitro Cytotoxicity

**Fig. 7:**
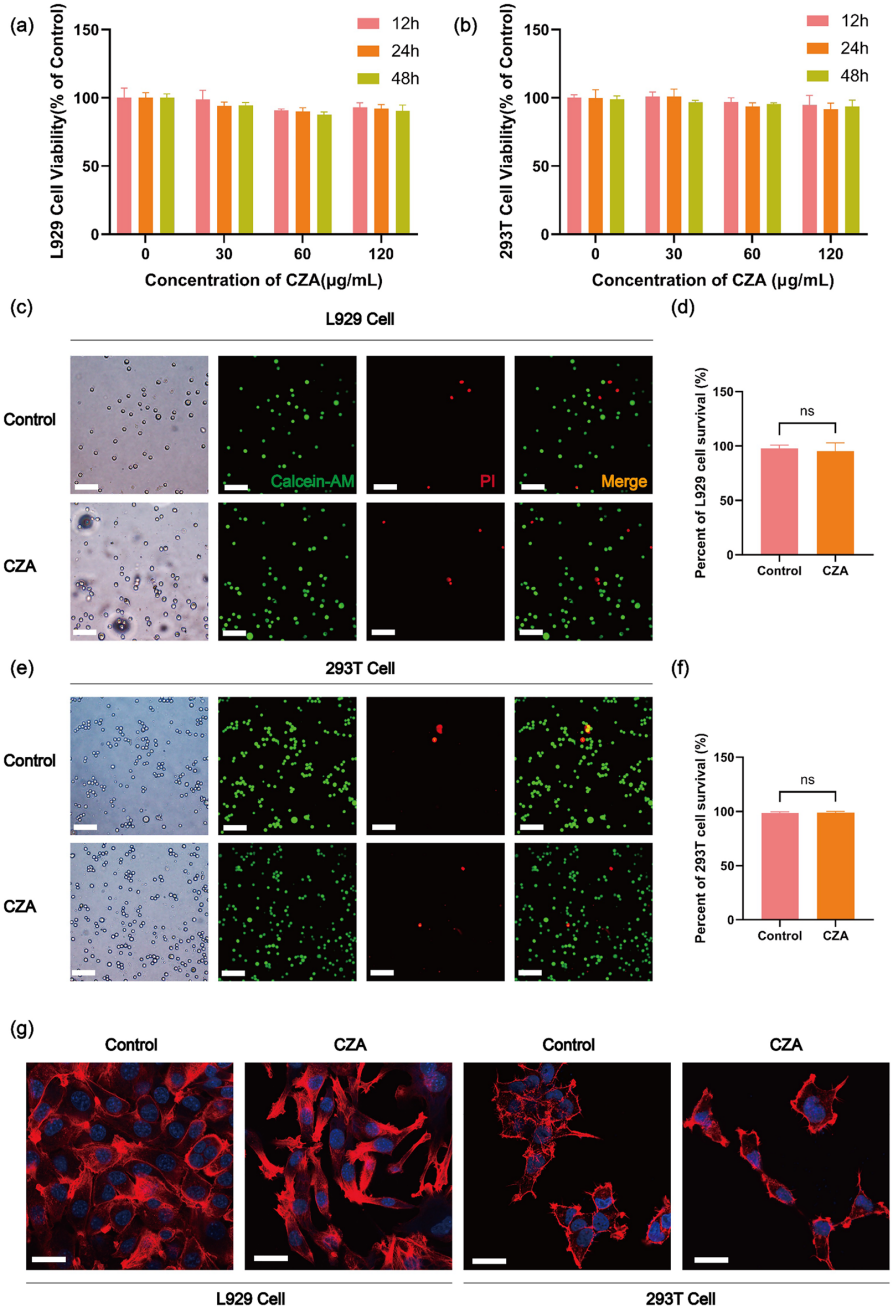
Material cytotoxicity data and live and dead cytoskeleton staining In vitro cytotoxicity and apoptosis investigation. **a** In vitro cytotoxicity assays for different concentrations and incubation times of CZA in L929 cells. **b** In vitro cytotoxicity assays for different concentrations and incubation times of CZA in 293T cells. **c** The apoptosis of L929 cells after CZA treatments was detected by calcein AM/PI staining (Green: live cells, Red: dead cells). Scale bars: 50 μm. **d** Statistical chart of L929 cell survival quantity. **e** The apoptosis of 293T cells after CZA treatments was detected by calcein AM/PI staining (Green: live cells, Red: dead cells). Scale bars: 50 μm. **f** Statistical chart of 293T cell survival quantity. **g** Cytoskeleton staining of L929 and 293T cells (Red: cytoskeleton, Blue: nucleus). Scale bars: 5 μm

To further investigate CZA cytotoxicity in vitro, we treated 293T cells and L929 cells at concentrations of 30, 60, and 120 μg mL^−1^ for 12, 24, and 48 h, respectively. Using the CCK-8 kit, it was observed that there was no significant decrease in cell viability of both L929 and 293T cell lines at 60 or 120 μg mL^−1^ even after 48 h of treatment (Fig. 7a, b), which suggested that CZA may not have significant cytotoxicity on cells. To further investigate whether CZA incubation leads to cell death, Calcein-AM and PI staining were performed on 293T cells and L929 cells incubated with CZA for 24 h (Fig. 7c–f). Cells that undergo necrosis or are in the final stage of apoptosis will be stained red by PI. Compared with the control group, there was no statistically significant difference in the cell survival rate of L929 cells or 293T cells incubated with CZA for 24 h. To observe whether CZA affects cytoskeleton of cells or not, the cytoskeleton of L929 cells or 293T cells incubated with CZA for 48 h was stained with phalloidin. No significant toxic effects of CZA treatment on the cytoskeleton of L929 cells or 293T cells were observed using laser confocal microscopy (Fig. 7g). The above results suggest that CZA may have acceptable biological safety.

### Evaluation of Antioxidant Ability In Vitro

**Fig. 8:**
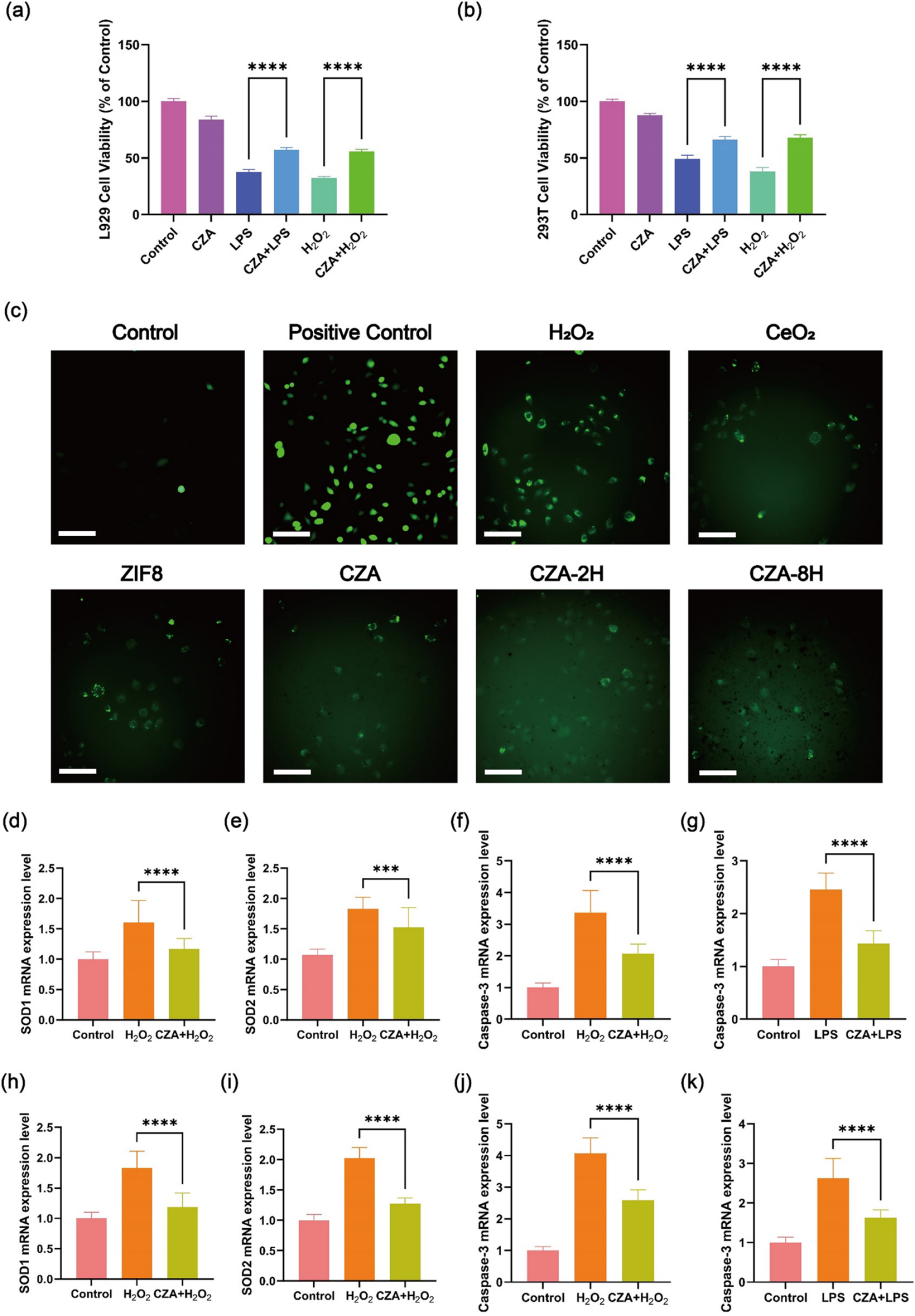
Anti-inflammatory data characterization of materials In vitro CZA enhancing cell antioxidant function investigation. **a** Cells viabilities of L929 cells after the treatment with PBS, CZA, LPS, CZA + LPS, H_2_O_2_, and CZA + H_2_O_2_. **b** Cells viabilities of 293T cells after the treatment with PBS, CZA, LPS, CZA + LPS, H_2_O_2_, and CZA + H_2_O_2_. **c** The images of H_2_O_2_ induced L929 cells with oxidized DCFH-DA fluorescence treated with different components. Scale bars: 50 μm. **d-f** SOD1, SOD2, Caspase-3 gene expression level of L929 cell after treated with H_2_O_2_ or CZA + H_2_O_2_. **g** Caspase-3 gene expression level of L929 cell after treated with LPS or CZA + LPS. **h–j** SOD1, SOD2, Caspase-3 gene expression level of 293T cell after treated with H_2_O_2_ or CZA + H_2_O_2_. **k** Caspase-3 gene expression level of 293T cell treated with LPS or CZA + LPS. (mean ± SD, n = 3; *P* values: *P < 0.05, **P < 0.01, ***P < 0.001)

To evaluate whether CZA significantly enhances cellular antioxidant or inflammatory capacity in vitro, we treated cells with H_2_O_2_ or LPS for 1 h after CZA pre-incubated for 24 h, and then measured cell viabilities (Fig. 8a, b). Compared with the non-pre-incubated group (just treated by H_2_O_2_ or LPS), the increased cell viabilities were observed in pre-incubated CZA + LPS L929 cells (41.42%), pre-incubated CZA + H_2_O_2_ L929 cells (55.61%), pre-incubated CZA + LPS 293T cells (27.28%) and pre-incubated CZA + H_2_O_2_ 293T cells (58.74%), which suggests that pre-incubation of CZA enhances the antioxidant or inflammatory abilities of L929 cells and 293T cells. We use ROS probe DCFH-DA to detect the production of reactive oxygen species in cells (pre-incubated with CeO_2_, ZIF8, CZA, CZA-2H, CZA-8H) treated with hydrogen peroxide. Fluorescence microscopy was used to observe of intracellular ROS probe luminescence in different treatment groups. Strong green fluorescence was observed in both group treated with H_2_O_2_ and ROS reagent that can cause strong oxidative stress in the kit (positive control), while in other group pre-incubated with CeO_2_, ZIF-8 or CZA, intracellular ROS levels decreased compared to the H_2_O_2_ group (Fig. 8c). In order to acidify CZA and release CeO_2_ to exert the antioxidant capacity of CZA, CZA soaked in PBS at pH 4–4.5 for 2 and 8 h, respectively. L929 cells were treated with H_2_O_2_ for 1 h after CZA (no soaking), CZA-2H, and CZA-8H pre-incubated 24 h. It was observed that CZA-8H can better reduce the production of ROS in L929 cells (Fig. 8c). This indicates that acid immersion can decomposition ZIF-8 framework and release encapsulated CeO_2_, leading to effectively remove ROS. In addition, if the material can promote cell migration and proliferation during the proliferative phase of wound healing, it will be very beneficial to promote wound healing. We further carried out the cell scratch experiment to investigate the ability of the material to promote cell migration after the effect of ROS scavenging. As shown in Fig. S4. compared with the control group, CZA NPs had certain promotion effect on cell migration. For a more intuitive demonstration, the healing rate of the scratched area was quantitatively calculated using the Image J software (Fig. S5). The data showed that the scratch mobility of CZA cultivated in cells for 24 h could reach 53%, which was almost reached the value of CeO_2_ NPs group. Cell scratch assay showed that CZA materials containing CeO_2_ NPs components can effectively promote cell migration, which is essential for wound healing. To understand how CZA helps cells reduce intracellular oxidative stress or resist inflammation, we further extracted mRNA from cells and evaluated transcriptional expression of antioxidant and apoptotic genes. Similar to previous experiments, cells were pre-incubated with CZA for 24 h or without pre-incubation, and then treated by LPS or H_2_O_2._ Compared with the control group, the upregulation of antioxidant related mRNA (SOD1 and SOD2) in the H_2_O_2_ treated L929 and 293T group showed activation of intracellular antioxidant mechanisms. However, in the CZA + H_2_O_2_ L929 and 293T group, the corresponding cellular expression levels of these mRNA were significantly down-regulated, which suggests that CZA helps cells remove ROS (Fig. 8d–i). After pre-incubation of CZA 24 h, the mRNA expression level of caspase-3 in L929 or 293T cells treated with H_2_O_2_ or LPS is lower than that in L929 or 293T cells not pre-incubated (Fig. 8f–k), which suggested that pre-incubation of CZA helps cells enhanced the anti-apoptotic ability. In addition, in order to better understand the performance of CZA in clearing ROS at the cellular level, we further extracted mRNA from cells and detected the expression levels of several inflammatory factors, including IL-6 and TNF-α. As shown in Fig. S6a, b. the levels of IL-6 and TNF-α in 293T cells were significantly increased after LPS was added, while the levels of inflammatory factors in cells were significantly decreased after CZA NPs treatment, indicating that CZA NPs can effectively alleviate inflammation.

### Wound Infection Treatment

**Fig. 9:**
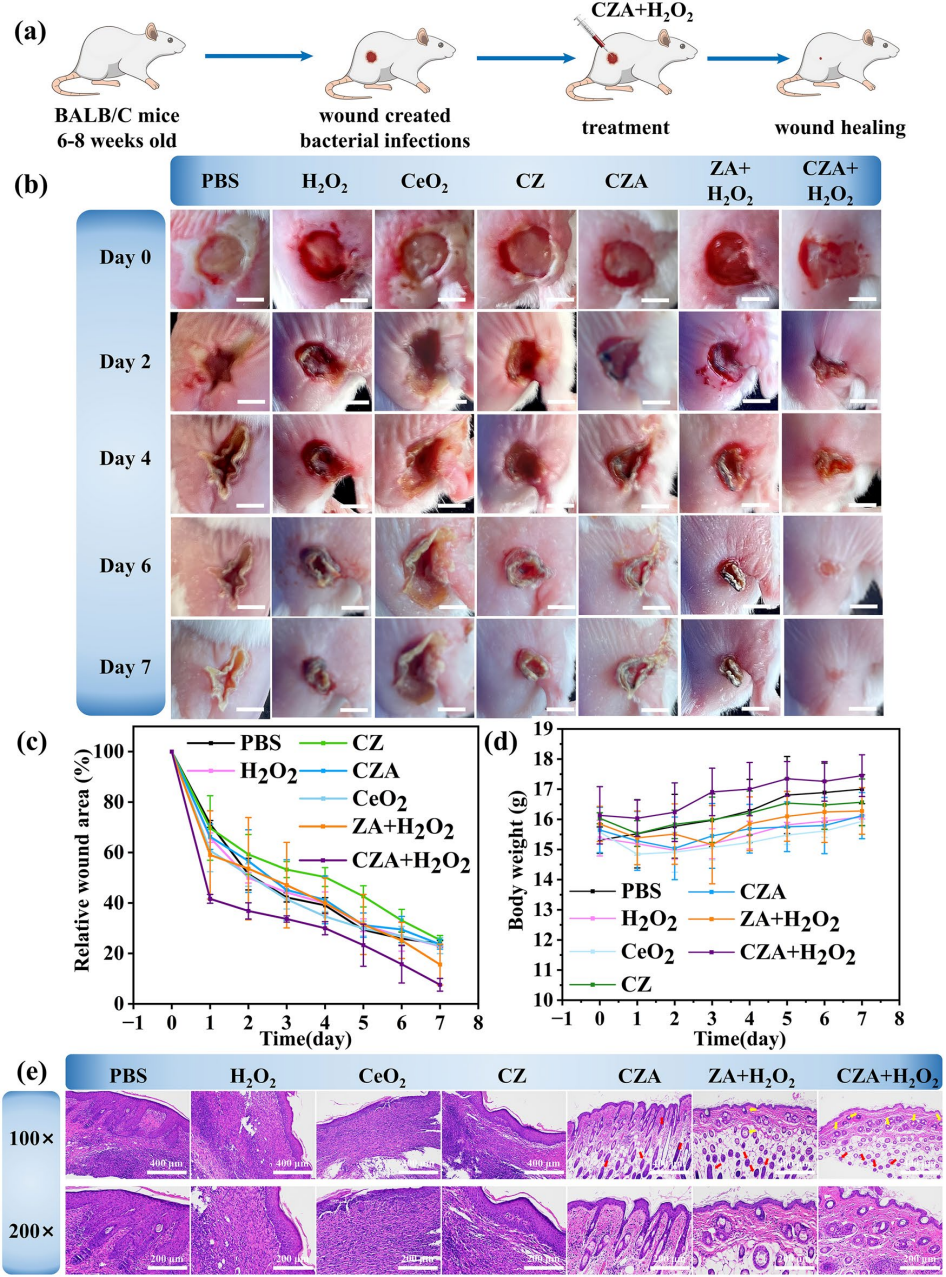
CZA antibacterial/promote the healing of animal models. **a** Schematic diagram of anti-infection implementation scenario in vivo for CZA. **b** Wound area graph treated differently along time points. Scale bars: 5 mm. **c** Corresponding changes in relative wound area. **d** Mice body weight. **e** H&E stained photographs at wound site (gules and yellow arrows mean hair follicles and blood vessels) (mean ± standard deviation, n = 3; *P* values: *P < 0.05, **P < 0.01, ***P < 0.001)

In order to deeply explore the anti-microbial effects of CZA NPs on wound healing, we subcutaneously injected BALB/c mice with 200 μL of 10^7^ CFU mL^−1^ *S. aureus*, as illustrated in Fig. 9a. Following a 24-h incubation period, a skin wound approximately 7 mm in diameter was formed at the injection site where the bacteria had been subcutaneously incubated. Stochasticly allotted infected mice into 7 sets of 3 per set, then handled with PBS, H_2_O_2_, CeO_2_, CZ, CZA, ZIF-8/Au (ZA) + H_2_O_2_, and CZA + H_2_O_2_, respectively. Using PBS treatment set as the control. Figure 9b, c presents digital photographs and the relative wound area with different treatments at various time periods (0, 2, 4, 6, and 7 days), with all mice exhibiting clear festering of the wounds on the first day. The mice exhibited a declining trend in weight during the first 1–3 days of infection (Fig. 9d), indicating that the bacterial infection to be worked. After treatment, the weight of mice began to show a steady upward trend all the time, indicating that after treatment, the material itself did not cause much harm to mice and had good biocompatibility. At the same time, the wound area of mice also showed a steady downward trend, especially in the first 3 days. Finally, on the seventh day, the wound area of mice treated with PBS, H_2_O_2_, CeO_2_, CZ, CZA, ZA + H_2_O_2_, CZA + H_2_O_2_ was reduced to 23.8%, 22.8%, 23.1%, 25.3%, 23.6%,15.6%, and 7.5% (Fig. 9c), respectively. Among them, Compared with ZA + H_2_O_2_ group, CZA + H_2_O_2_ group showed the better therapeutic effect. The above results indicate that the catalytic capacity of CZA plays a vital role in antibacterial therapy and demonstrated that the ROS scavenging function of CeO_2_ NPs played an additional promoting effect on wound healing after the completion of ROS antimicrobial treatment.

**Fig. 10:**
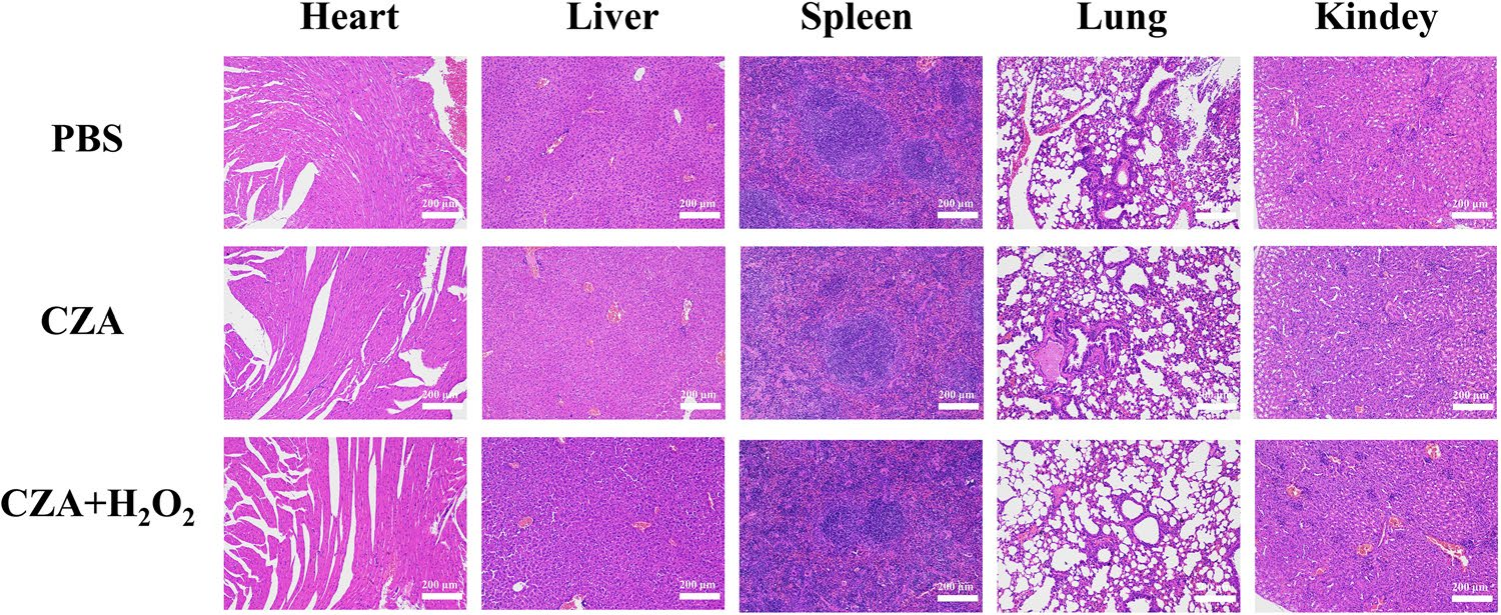
Images of H&E-stained heart, liver, spleen, lung, and kidney obtained from mice of PBS, CZA, and CZA + H_2_O_2_ groups

In order to obtain more reliable details and further appraise the wound healing process, we took skin tissues from the wound and subjected to H&E staining, as shown in Fig. 9e. Consistent with the digital photographs, glucose-treated mice exhibited partial skin tissue regeneration and some subcutaneous hemorrhage, stating that the wound was not fully closed. The tissue regeneration of mice in PBS, ZIF-8, CZ, CZA, and ZA was similar and the tissue structure was good. Abundantly distributed capillaries provide nutrients, indicating rapid regeneration of skin tissue. In contrast, CZA and H_2_O_2_-treated mice showed good skin structure and reduced capillaries, indicating utter regeneration of the wound site. In addition, the histopathological photographs in Fig. 10 revealed that the major organs of infected or treated mice were not conspicuously damaged. In summary, based on the appraisement of wound bactericidal properties, CZA NPs exhibited the ability of bacterial removal and wound healing by effectively killing bacteria and accelerating the healing process of infected wounds in the presence of H_2_O_2_. In addition, CZA NPs exhibit good biocompatibility, making them promising candidates for the treatment of infected wounds.

## Conclusions

We designed a dual synergetic MOF nanozyme (CeO_2_@ZIF-8/Au), which possesses both antibacterial and ROS regulating functions for wound repair. Firstly, it can catalyze and product ·OH to complete the efficient antibacterial effect to kill bacteria. Then, as the ZIF-8 structure decomposes in the acidic microenvironment, the CeO_2_ core is gradually released, which can effectively remove ROS and auto-regulate ROS balance, realize the purpose of reducing inflammation and promote wound healing. The innovation of this work mainly lies in the auto-regulation of ROS balance effective by combination of ROS antibacterial and ROS scavenging anti-inflammatory functions with CeO_2_@ZIF-8/Au nanoplatform, which can not only achieve high antibacterial efficiency, but also promote wound healing, and provide a new idea for the nano-catalytic system in the field of wound healing of bacterial infection. Besides, such innovative ROS spontaneous regulators hold immense potential for revolutionizing the field of cancer therapies [49–58], anti-SARS-CoV-2 and other viruses [59–61], and much broader biomedical applications due to the fundamental importance of ROS in health regulation.

## Supplementary Information

Below is the link to the electronic supplementary material.

Supplementary file1 (PDF 816 kb)
