# Supplementary material for: ROS Balance Autoregulating Core–Shell CeO2@ZIF-8/Au Nanoplatform for Wound Repair
Source: Nanomicro Lett. 2024 Mar 21;16:156. doi: 10.1007/s40820-024-01353-0 (PMC10957853; doi:10.1007/s40820-024-01353-0)
Supplement: Supplementary file 1 — Supplementary file1 (PDF 816 kb) [file 40820_2024_1353_MOESM1_ESM.pdf]

Supporting Information for

ROS Balance Autoregulating Core-Shell CeO_2@ZIF-8/Au Nanoplatform for Wound Repair

Xi Zhou [1], Quan Zhou [1], Zhaozhi He [1], Yi Xiao [3], Yan Liu[3], Zhuohang Huang [1], Yaoji Sun [2], Jiawei Wang [2], Zhengdong Zhao[4], Xiaozhou Liu[4], Bin Zhou [5], Lei Ren [1], Yu Sun[4, *], Zhiwei Chen [2, *], Xingcai Zhang [3, *]

[1] The Higher Educational Key Laboratory for Biomedical Engineering of Fujian Province, Research Center of Biomedical Engineering of Xiamen, Department of Biomaterials, College of Materials, Xiamen University, Xiamen, 361005, People's Republic of China

[2] Department of Electronic Science, Fujian Provincial Key Laboratory of Plasma and Magnetic Resonance Research, School of Electronic Science and Engineering, Xiamen University, Xiamen 361005, People's Republic of China

[3] John A Paulson School of Engineering and Applied Sciences, Harvard University, Cambridge, MA, 02138, USA

[4] Department of Otorhinolaryngology, Union hospital of Tongji medical college, Huazhong University of Science and Technology, Wuhan, 430022, People's Republic of China

[5] NO.1 Middle School affiliated to Central China Normal University, Wuhan, 430223, People's Republic of China

*Corresponding authors. E-mail: zhangxingcai@wteao.com or xingcai@seas.harvard.edu (Xingcai Zhang); chenzhiwei@xmu.edu.cn (Zhiwei Chen); sunyu@hust.edu.cn (Yu Sun)

Supplementary Figures

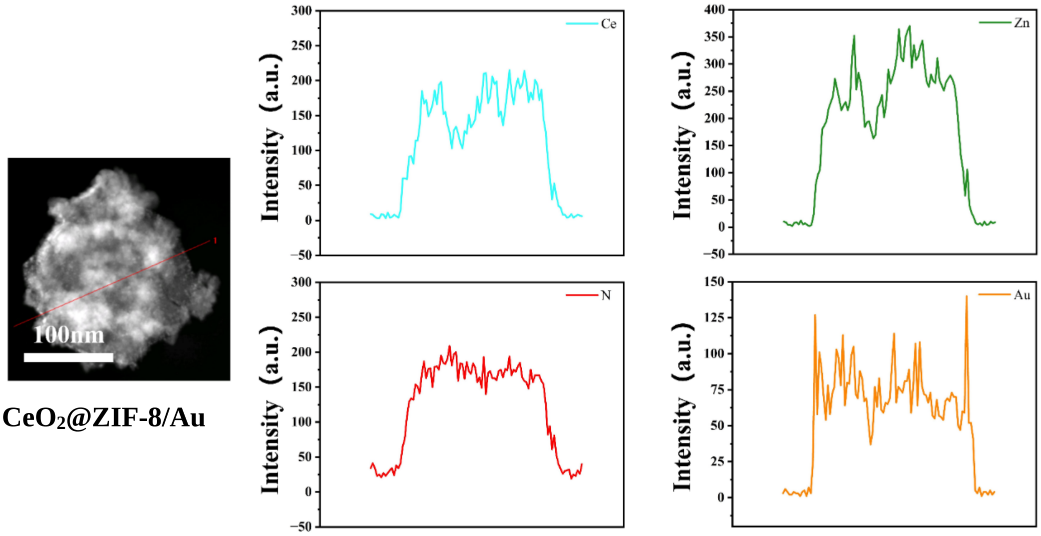

Fig. S1 Line distribution of elements in CZA(Ce、Zn、N、Au)

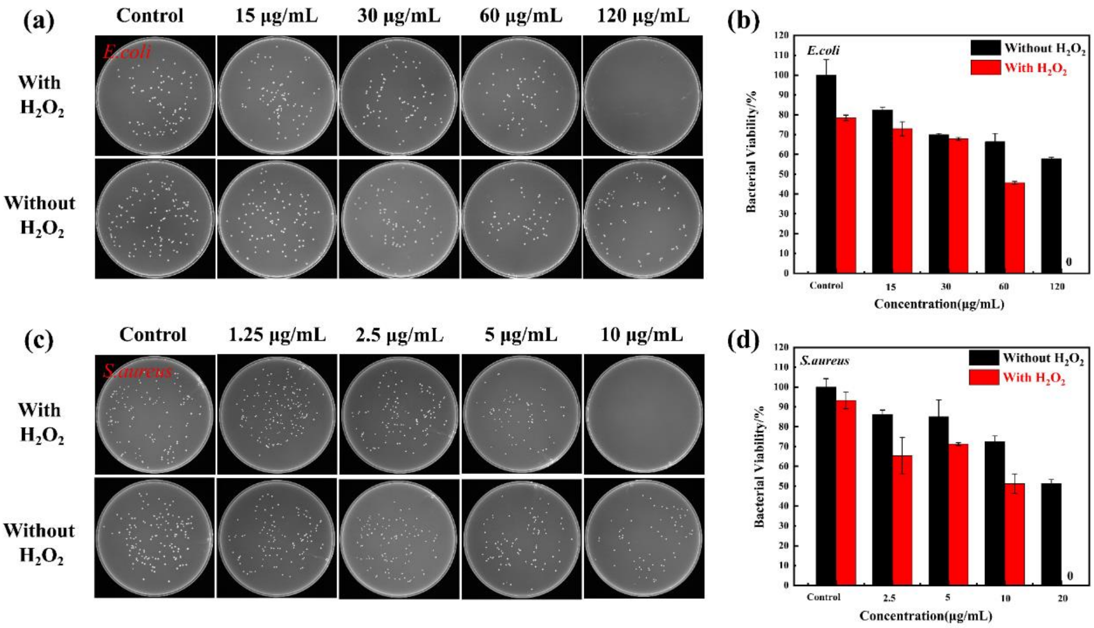

Fig. S2 Agar plate photographs for **a** E. coli and **c** S. aureus treated with different concentrations of CeO_2@ZIF-8/Au(CZA) with or without the addition of H_2O_2 and **b, d** the corresponding statistical number of colonies respectively

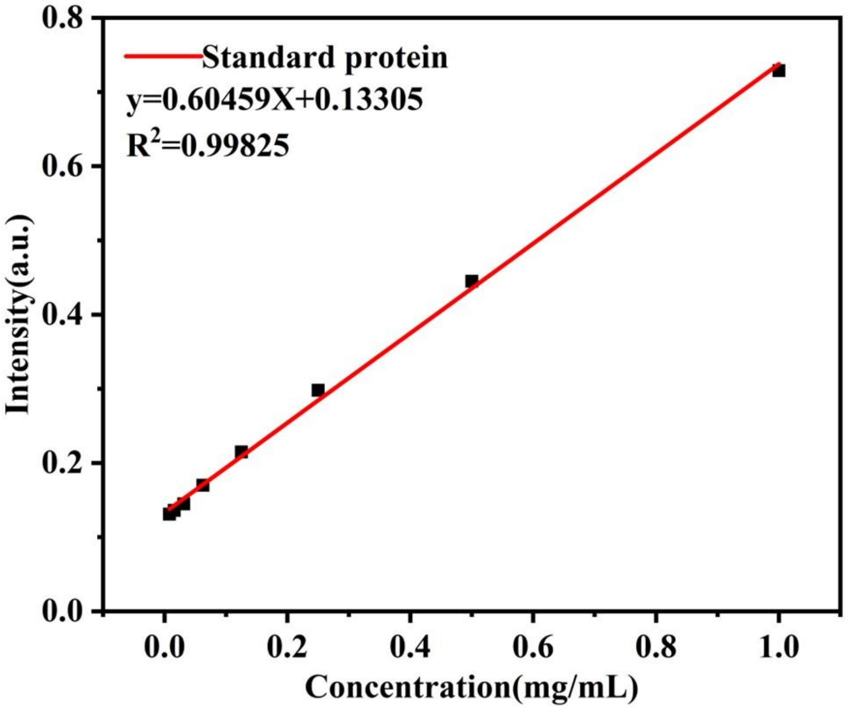

Fig. S3 Standard curve of the standard protein applied in BCA protein test

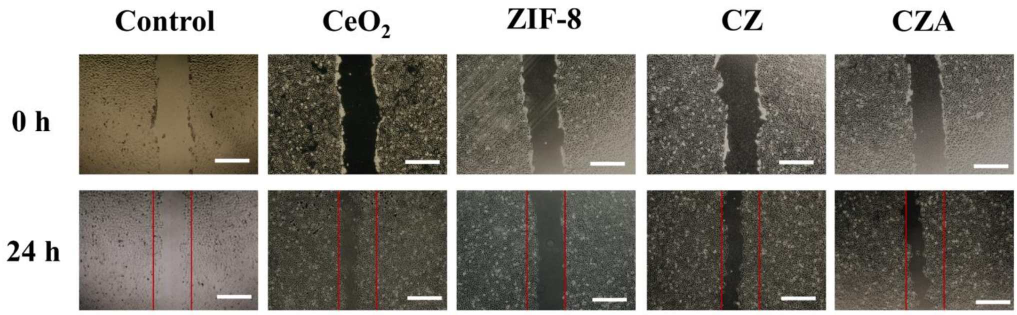

Fig. S4 Photographic images of HUVECs migration with different treatments (Scale bar: 200 μm)

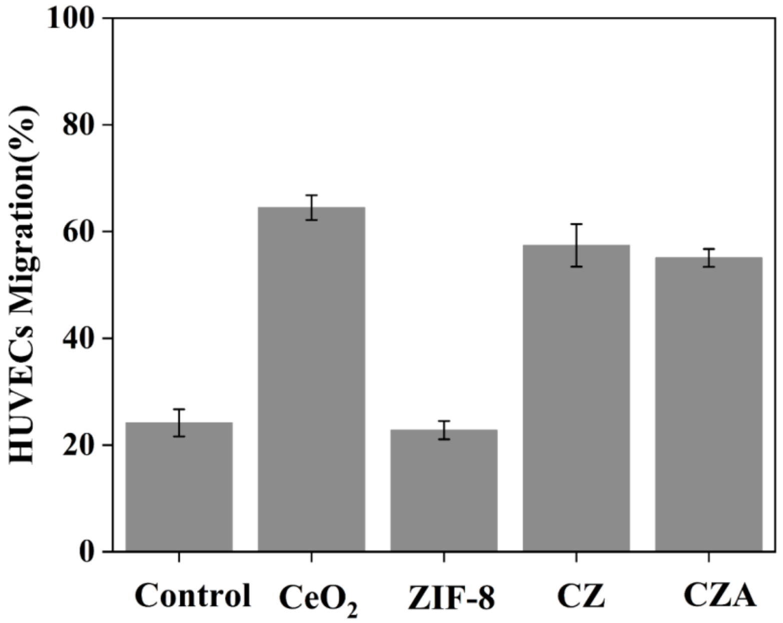

Fig. S5 HUVECs migration with different treatments, calculated by Image J

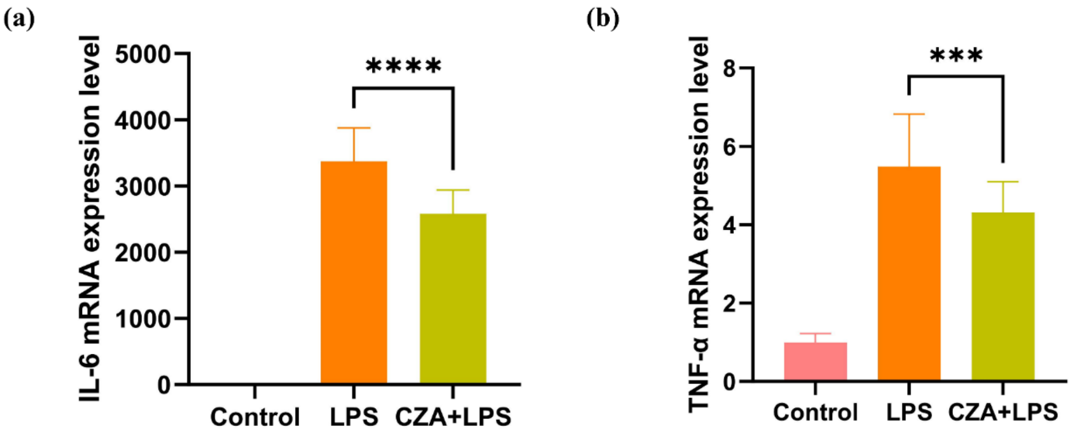

Fig. S6 IL-6 **a** and TNF-α **b** gene expression level of 293T cell after treated with LPS or CZA +LPS
